# Cervical Necrotizing Fasciitis

**DOI:** 10.1590/0037-8682-0379-2023

**Published:** 2023-09-22

**Authors:** Suzan Şahin

**Affiliations:** 1 Dr. Lütfi Kırdar Kartal City Hospital, Department of Infectious Diseases and Clinical Microbiology, İstanbul, Turkey.

A previously healthy 21-year-old man presented with a one-week history of generalized malaise, fever, and erythematous neck swelling with central black discoloration (Figure 1A). He appeared cachectic and had a fever (39.1 ºC). Physical examination revealed a foul-smelling, diffused erythematous edema on the neck’s right side, featuring a necrotic area measuring 10 × 10 cm. His white blood cell count was 23,600/mm^3^, C-reactive protein level was 175 mg/L, erythrocyte sedimentation rate was 71 mm/h, alanine aminotransferase level was 63 U/L, and aspartate aminotransferase level was 53 U/L. The patient was admitted to the otolaryngology clinic, where empirical antibiotic therapy was initiated, comprising 1 g each of vancomycin and meropenem administered twice and thrice daily, respectively. 

**FIGURE 1A:**
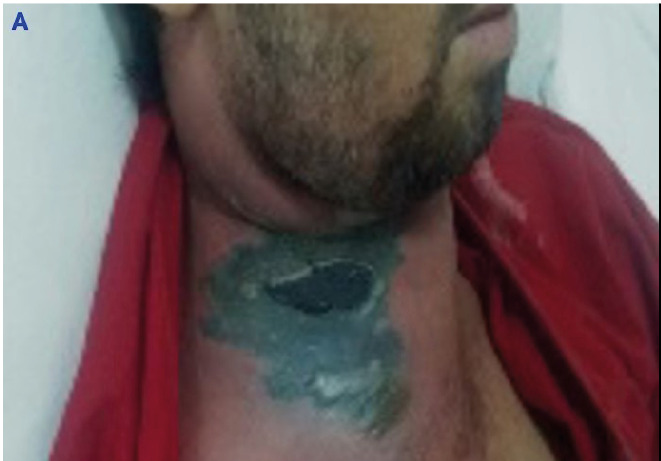
Erythematous swelling of the neck with black discoloration in the middle.

Neck ultrasonography showed an abscess, and computed tomography showed a large right-sided fluid-density lesion. This lesion, interspersed with air patches, extended to the supraclavicular region (Figure 1B). Despite the persistent fever, the abscess was drained. By the second day of hospitalization, the patient’s fever had subsided, and there was a partial improvement in his clinical condition. Cultures from both the blood and the abscess showed no pathogenic growth. Laboratory findings normalized, and histopathologic examination revealed acute suppurative necrotizing inflammation. Consequently, a diagnosis of necrotizing fasciitis was established. 

**FIGURE 1B:**
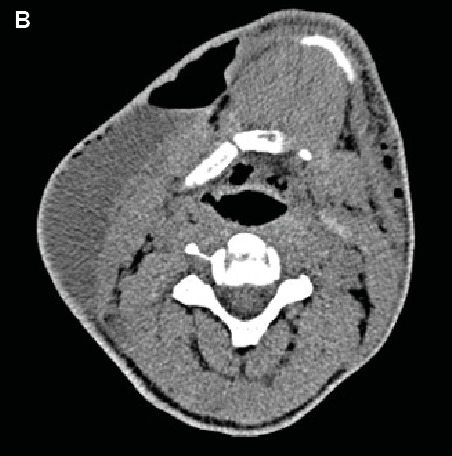
A CT scan shows a large lesion of fluid density on the right side of the neck.

Necrotizing fasciitis, a rare and life-threatening bacterial infection, necessitates immediate identification and treatment to prevent fatality^1^. Early administration of antibiotics and drainage are particularly crucial^2^. After his symptoms subsided (Figure 1C), the patient was referred to the plastic surgery department for skin grafting.

**FIGURE 1C:**
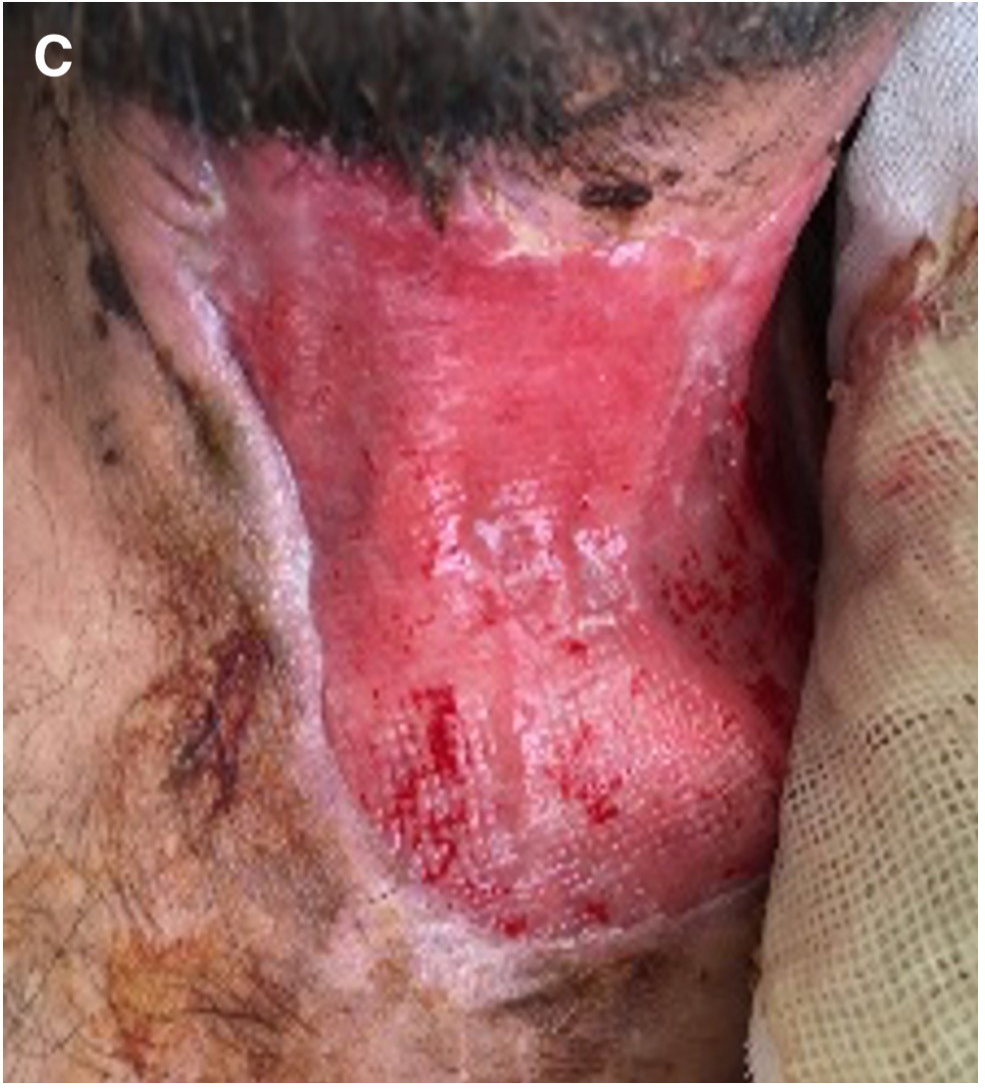
The resolved lesion after antibiotic therapy and drainage.
